# Integration of Albanian nurses in Germany: Employment challenges and opportunities - A descriptive study

**DOI:** 10.3934/publichealth.2025023

**Published:** 2025-04-08

**Authors:** Alketa Dervishi, Simon Jäger, Blerina Duka, Etleva Kika, Valbona Bezhani, Ardit Lena, Dhurata Ivziku

**Affiliations:** 1 Faculty of Technical Medical Sciences, University of Medicine, Tirana, Albania; 2 Department of Nursing, RE-ALIS GmbH, Speyer, Germany; 3 Faculty of Medicine, University “Our Lady of the Good Counsel”, Tirana, Albania; 4 Directory of Teaching, Future Center, Tirana, Albania; 5 Department of Health Professions, Fondazione Policlinico Universitario Campus Bio-Medico, Rome, Italy

**Keywords:** nurses, human migration, social integration, work integration, Albania nurses, Germany, nursing workforce, challenges, opportunities, human capital

## Abstract

Nurse migration from low- and middle-income countries to high-income nations is a significant global phenomenon. This study aimed to examine the opportunities and challenges faced by Albanian nurses during their work integration after migrating to Germany. A descriptive cross-sectional quantitative study was conducted using convenience sampling with a snowball recruitment technique. A total of 162 Albanian nurses working in Germany, mostly female and married, participated in the survey. The findings revealed that the overall integration process was satisfactory, particularly regarding the recognition of professional qualifications, respect from colleagues, patients, and families, career opportunities, and autonomy at work. The host institutions supported integration through mentorship programs, language training, procedural training, and education on the German healthcare system. However, nurses reported challenges in medical terminologies, telephone communication, and healthcare documentation. The participants expressed the need for more comprehensive pre-departure information to facilitate smoother transitions. The study highlights the pivotal role of managers and institutions in the integration process and in creating inclusive and equitable work environments. Germany serves as a model for implementing effective integration activities for migrant nurses. Albania must adopt stronger strategies to retain nurses within the country and enhance its human capital by improving the working conditions and providing career development opportunities. Future research is recommended to explore the integration processes of Albanian nurses across other European countries. Subsequent studies should concentrate on identifying and addressing potential barriers to integration while fostering opportunities for collaboration between the origin and destination countries. Such research can contribute to academic, political, and social frameworks that enhance the migration experience for migrant nurses.

## 1. Introduction

Nurse migration from low- and middle-income countries to high-income nations is a significant global phenomenon. The migration worsens the projected shortfall of 10 million health workers in those countries by 2030 [1]. According to a report by the World Health Organization (WHO), the Organization for Economic Cooperation and Development (OECD), and the International Labor Organization (ILO), international nurse migration has significantly risen in recent years [2]. Currently, 15% of healthcare workers (one in every 8) are employed in a country different from where they were born or received their training [3]. Wealthier countries actively recruit nurses from low- and middle-income nations to address their staffing shortages, which often leads to staff deficits in the source countries [4]. The international recruitment and migration process must be ethical and regulated to benefit both the destination and origin countries [4].

Emigration concerns of the nursing profession is a pressing national issue for Albania. Over the last three decades, the country has witnessed a substantial exodus of its 18–40-year-old population [5], ranking the country among the top nations globally for migration patterns [6]. The pattern of Albanian migration reveals that most (74.6%) migrate to European Union (EU) countries and 19.1% migrate to North America [7]. Within Europe, the migration trends have shifted from traditional destinations such as Italy and Greece toward North-Western countries such as Germany, the United Kingdom, Sweden, Norway, Switzerland, and Austria [7].

For Albanian nurses, Germany has emerged as the top destination [8], driven by a combination of Germany's targeted recruitment policies, efforts to support the transition process, and the widespread teaching of the German language in Albanian schools [7]. Existing research has predominantly focused on Albanian nurses' migration aspirations rather than their lived experiences in the host countries. This research seeks to close this gap by exploring the opportunities and challenges Albanian nurses face in their professional integration in Germany.

Albania, a middle-income country in the southeast Mediterranean Region, organizes its nursing education through a Bachelor's university degree program aligned with Level 6 of the European Qualification Framework (EQF) [9]. To practice as a nurse, individuals must pass a licensing examination and register with the Albanian Order of Nurses (UISH) [10]. As of October 2024, UISH data reports that Albania has 25,305 registered nurses [11],[12], who are unevenly distributed across the country [13]. UISH actively monitors nurse mobility and has reported that over 6000 licensed nurses migrated abroad between 2016 and 2024, reflecting an approximate 200% growth in migration requests in the last few years [8]. In addition, a recent study documented a desire to migrate among 83% of registered nurses [7]. These trends highlight a substantial challenge for Albania's healthcare system, emphasizing the need for strategies to retain nursing professionals and address workforce sustainability.

Recent research in the country has explored motivations that lead Albanian healthcare professionals to emigrate. For example, Krasniqi et al. [14] surveyed 1037 healthcare professionals in Albania, finding that 62.7% of participants expressed a desire to migrate abroad, with this inclination being particularly pronounced among younger professionals aged 25–34 [15]. The key drivers of these migration aspirations include better salaries (38.6%), career advancement opportunities (28.8%), and improved working conditions (24.1%) [14]. Additionally, 48.3% of the respondents identified the standard of living and economic stability as critical factors that influenced their decision [14]. These findings underscore Albania's urgent need to retain its healthcare workforce and address this pressing challenge. Indeed, the Albanian government is actively implementing various policies to create favorable conditions to prevent the emigration of professionals. These measures include salary increases funded by the government, significant investments in hospitals and health centers, improvements in working conditions, the adaptation of clinical protocols, expanded opportunities for training and continuing education, and recruitment strategies [15],[16].

In response to the nursing staff crisis, Western countries have implemented policies and allocated resources to attract migrant nurses and streamline their entry processes. Since 2012, Germany has implemented numerous comprehensive measures to regulate, recruit, and facilitate integration programs for migrants, thus establishing itself as an attractive destination for nurses [17],[18]. Key strategies include simplifying the migration processes for qualified professionals from non-EU countries, accelerating visa processing systems, and offering enhanced language proficiency courses [19]. Additionally, the German government and hosting institutions support social and professional integration through specialized training, advisory services, an improved access to housing and social networks, administrative assistance, mentorship, and intercultural training [19],[20]. In the countries of origin, the mobilization, selection, and initial preparation of prospective migrants and recruitment initiatives are often facilitated by labor market intermediaries, such as placement agencies and care institutions [19]. Moreover, autonomous recruitment efforts are conducted through networks of universities and language schools [21]. These combined efforts aim to address the staff shortages in the country while ensuring the successful integration of foreign healthcare workers.

The existing literature on migrant nurses in Germany is limited. While most studies utilize qualitative designs to explore the experiences of migrant nurses, empirical quantitative research remains scarce [22]. Therefore, this study aims to address this gap by examining the opportunities and challenges Albanian nurses face during their work integration after migrating to Germany.

The findings from this study are essential to understand the global phenomenon of nurse migration, as they contribute to the development of supportive frameworks and effective strategies to manage diversity and enhance the well-being of nurses in host countries. Additionally, the results will provide invaluable guidance to Albanian nurses considering migrating to Germany, thereby equipping them with a clearer understanding of their opportunities and challenges.

## 2. Materials and methods

### 2.1. Study design

The study used a descriptive observational cross-sectional design and followed the Strengthening the Reporting of Observational Studies in Epidemiology (STROBE) guidelines [23].

### 2.2. Setting and sampling

A convenience sampling strategy combined with a snowball recruitment technique was employed [24],[25]. The researchers initially reached out to former nursing students working in Germany and provided them with detailed information about the study's objectives, the inclusion and exclusion criteria, and the type of data being collected. Eligible individuals were invited to participate and were also encouraged to share information about the study with others in their professional or social networks who met the inclusion criteria [25]. This approach was particularly effective in increasing the participation within this hard-to-reach group. Participation in the study was entirely voluntary, thus allowing individuals to freely decide whether to take part. Data collection was conducted in December 2024 using an online survey.

#### 2.2.1. Inclusion and exclusion criteria

The study included Albanian nurses who migrated to Germany within the last five years and are currently employed as registered nurses in hospital settings. The exclusion criteria encompassed nurses who migrated to Germany more than five years ago, those working in residential, homecare, rehabilitation, or community care settings, as well as individuals who were employed as nursing assistants, under alternative work contracts, or awaiting the recognition of their professional qualifications.

### 2.3. Data collection instrument

Based on the literature review, the research team developed a list of survey items. Subsequently, in two discussion sessions with two UISH nurse leaders and four nurse educators, each with over 10 years of experience. The explored areas were reviewed leading to the finalization of the survey. The survey was distributed to 10 final-year nursing students and 10 nurses to gather their feedback on the clarity of the items and the response options. No modifications were recommended.

Data were collected through an online survey, in Albanian, using a structured questionnaire with closed-ended questions specifically designed to capture key information on the opportunities and challenges faced by migrant nurses during their integration process across several critical areas. At the beginning of the survey, the participants were provided with detailed information about the study's aims, the privacy measures, and the data handling procedures. Then, they were asked to provide informed consent. Participation was entirely voluntary, and the participants had the option to either complete the entire survey or to only respond to selected parts.

The survey consisted of a total of 18 items, beginning with demographic questions. The participants were asked to provide information about their gender, living location of origin (urban or rural), family status, highest level of nursing education, and years of employment in Germany. Subsequently, in 6 items, the survey explored the participants' perceptions of their integration within the workplace. This section evaluated aspects such as their career opportunities, the extent to which their professional qualifications were respected by staff, patients, and families, the level of autonomy granted by managers compared to the local staff, their knowledge of institutional policies and procedures, and their overall perception of being treated with respect in the workplace, as comparable to local staff. All items were rated on a scale from 1 (completely disagree) to 4 (completely agree). Additionally, the participants were asked whether they had experienced different treatments at work as a nurse due to language barriers, with response options of 0 (no) and 1 (yes).

In the second section (3 items), the participants were asked about their German language abilities and its impact on their nursing practice. They were instructed to only select one option from a list of activities they found most challenging due to language barriers. The options included patient education, patient assessment, caregiver education, providing support to distressed patients, communication with physicians, nursing care documentation, telephone communication, and understanding patients. Additionally, the participants were asked to rate the overall level of difficulty they experienced with medical or professional German on a scale from 0 (not at all) to 10 (completely).

The third section (4 items) focused on the participants' experiences during their transition to life in Germany. The participants were asked whether their expectations for working in Germany were met, with response options ranging from 0 (no), 1 (yes), and 2 (partially). Additionally, they were asked if the information provided in Albania prior to migration was sufficient, with options of 1 (yes) and 0 (no). Furthermore, the participants were asked about the resources offered for their integration upon their arrival in Germany. These included mentors, orientation programs, clinical skills evaluations, technology training, specific unit-based training, and information about the German healthcare system. Additionally, from the same list, they were asked to select the resources they believed would have made their transition to Germany easier, if provided.

The survey was created using Google Forms, and the link was distributed to the interested participants via email and social media platforms, thus ensuring both anonymity and privacy.

### 2.4. Data analysis

Descriptive statistics were employed to summarize the demographic data and survey responses. Continuous variables were assessed for normality and reported as means with standard deviations (*SD*) when normally distributed or as medians with interquartile ranges (*IQR*) for non-normally distributed data. Ordinal and nominal variables were presented as frequencies and percentages. The relationships between variables were analyzed using Spearman's correlation Rho coefficients. The missing data for each item were evaluated but not replaced in the analysis. Instead, they were reported in the results to provide transparency on data completeness. All statistical tests were two-tailed, with a significance level set at *p* < 0.05. A data analysis was performed using the SPSS software, version 27.0 (IBM Corp., Armonk, NY, USA, 2019).

### 2.5. Ethics approval of research

The research adhered to the principles outlined in the Declaration of Helsinki [26] and complied with all current legislation governing clinical trials. The study was approved by the Ethics Committee of the University of Medical Sciences, Tirana, Albania with the protocol number n 3297.

## 3. Results

Overall, 162 surveys were received from Albanian migrant nurses in Germany. Missing data accounted for less than 5% of the total responses; therefore, their impact on the overall findings is minimal and does not compromise the validity or reliability of the results.

### 3.1. Sample characteristics

The survey responses were predominantly provided by female nurses (70.4%), with the majority being married (60.5%), working in Germany for less than one year (45.1%), and holding a bachelor's degree (58%). For additional details, refer to Table 1. These findings suggest that Albanian nurses migrating abroad are primarily female and married.

**Table 1.** Characteristics of the sample (*N* = 162).

Variable	*N* (%)
**Sex**	
Female	114(70.4)
Male	46 (28.4)
Missing	2 (1.2)
**Living origin**	
Urban	101(62.3)
Rural	56 (34.6)
Missing	5 (3.1)
**Family status**	
Single	61 (37.7)
Married	98 (60.5)
Missing	3 (1.9)
**Highest level of education**	
Bachelor degree	94 (58.0)
Clinical master certification	40 (24.7)
Master of Science Degree	24 (14.8)
Missing	4 (2.5)
**Tenure in Germany**	
<1 year	73 (45.1)
1 year	27 (16.7)
2 years	12 (7.4)
>2 years	43 (26.5)
Missing	7 (4.3)

### 3.2. Integration in the workplace

Workplace integration was perceived very positively by the participants. Approximately 72% agreed that they had similar career opportunities as their German peers, 79% reported overall respect in the workplace, 74% felt respected by staff, patients, and families, and 88% indicated they were granted a similar autonomy at work compared to their local colleagues. For additional details, refer to Table 2.

**Table 2.** Integration in Germany (*N* = 162).

Variable	*N* (%)
**Integration in the workplace**	
**Similar career opportunities**	
Completely disagree	22 (13.6)
Disagree	19 (11.7)
Agree	77 (47.5)
Completely agree	40 (24.7)
Missing	4 (2.5)
**Professional qualifications respected by staff**	
Completely disagree	15 (9.3)
Disagree	16 (9.9)
Agree	83 (51.2)
Completely agree	45 (27.8)
Missing	3 (1.9)
**Professional qualifications respected by patients and family members**	
Completely disagree	10 (6.2)
Disagree	6 (3.7)
Agree	84 (51.9)
Completely agree	60 (37.0)
Missing	2 (1.2)
**Autonomy granted by managers**	
Completely disagree	8 (4.9)
Disagree	11 (6.8)
Agree	73 (45.1)
Completely agree	68 (42.0)
Missing	2 (1.2)
**Knowledge of institutional policies and procedures**	
Completely disagree	14 (8.6)
Disagree	17 (10.5)
Agree	91 (56.2)
Completely agree	36 (22.2)
Missing	4 (2.5)
**Being treated with respect in the workplace**	
Completely disagree	19 (11.7)
Disagree	14 (8.6)
Agree	78 (48.1)
Completely agree	46 (28.4)
Missing	5 (3.1)

### 3.3. Language challenges in the workplace

A significant proportion of participants (45.7%) reported experiencing linguistic barriers in the workplace, with a moderate level of difficulty related to technical-medical language (mean score: 4.8 on a scale of 0 to 10); refer to Table 3 for details. The most challenging activities reported were telephone communication (30.2%), nursing care documentation (28.4%), and communication with physicians (11.7%). In close succession to the top three challenges, the respondents identified understanding patients (10.5%) and caregiver education (7.4%) as significant challenges.

**Table 3.** Linguistic challenges in Germany (*N* = 162).

Variable	*N* (%)
**Language abilities in the workplace**	
**Different treatment due to language barrier**	
No	86 (53.1)
Yes	74 (45.7)
Missing	2 (1.2)
**Level of difficulty experienced with medical/professional German**	
*Mean* (*SD*) (range 0–10)	4.8 (2.3)
Missing	4 (2.5)
**Challenging activities due to language competency**	
Telephone communication	49 (30.2)
Nursing care documentation	46 (28.4)
Communication with physicians	19 (11.7)
Understanding patients	17 (10.5)
Caregiver education	12 (7.4)
Support to distressed patients	9 (5.6)
Patient assessment	1 (0.6)
Patient education	1 (0.6)
Missing	8 (4.9)

### 3.4. Transition experience

The participants indicated that their expectations during the transition to Germany were either fully met (48.1%) or partially met (44.4%). A majority (92%) expressed a desire for additional information prior to migration, particularly about the German healthcare system (33.3%). In this section of the survey, the participants were asked to identify the primary resource available to them in their workplace by selecting only one option. As reflected in the responses, institutional support primarily included information about the German healthcare system (24.7%), mentorship programs (27.8%), and orientation programs (16.7%). The relatively low percentages across all categories may be attributed to the survey design, which limited the participants to select the most prevalent resource rather than multiple sources of support they may have received. For further details, refer to Table 4.

**Table 4.** Transition experience (*N* = 162).

Variable	*N* (%)
**Expectations for working in Germany were met**	
Yes	78 (48.1)
Partially	72 (44.4)
No	9 (5.6)
Missing	3 (1.9)
**Information provided in Albania prior to migration was sufficient**	
No	149 (92.0)
Yes	10 (6.2)
Missing	3 (1.9)
**Resources offered for the integration upon arrival in Germany**	
Mentors	45 (27.8)
Information about the German healthcare system	40 (24.7)
Orientation programs	27 (16.7)
Specific unit-based training	21 (13.0)
Technology training	13 (8.0)
Clinical skills evaluations	10 (6.2)
Missing	6 (3.7)
**Resources that would have made the transition to Germany easier**	
Information about the German healthcare system	54 (33.3)
Orientation programs	26 (16.0)
German language training	25 (15.4)
Mentors	18 (11.1)
Specific unit-based training	16 (9.9)
Clinical skills evaluations	8 (4.9)
Cultural training	2 (1.2)
Technology training	2 (1.2)
Nothing to suggest	11 (6.8)

### 3.5. Variables associated with the integration experience

We explored possible associations between the integration process and the other variables using the Rho of Spearman correlation coefficient. Correlations between the nurses' demographic variables and the integration experience were generally low and ranged from 0.18 to 0.21. The results suggest that the integration process is more positive for participants with family responsibilities, as they experience greater respect and autonomy at work (*r* = 0.21 and *r* = 0.20). Additionally, the level of autonomy given by the manager improves with tenure in the country (*r* = 0.19). Various significant correlations were found between the integration explored aspects and the integration experience, with the correlation coefficients ranging from 0.16 to 0.30. Language proficiency was the most influential factor in a positive integration experience, particularly in terms of respect and autonomy in the workplace. Conversely, a limited knowledge of the technical language (*r* = −0.17–−0.27) and unmet expectations (*r* = −0.18–−0.28) negatively impacted the respect, autonomy, and career opportunities. For further details, see Table 5.

**Table 5.** Associations between aspects explored and integration (*N* = 162).

Project	Career opportunities	Respect from staff	Respect patient/family	Autonomy from leader	Knowledge of policies	Overall respect
**Nurse demographic characteristics**						
family status	0.182	**0.007**	0.224	**0.007**	0.477	**0.012**
living origin	0.077	0.280	**0.025**	0.102	0.066	0.909
tenure in Germany	0.204	0.707	0.088	**0.020**	0.486	0.276
sex	0.647	0.770	0.893	0.308	0.858	0.554
level of education	0.833	0.734	0.640	0.424	0.408	0.121
**Integration aspects**						
difficulty technical language	**0.029**	**0.045**	**<0.001**	**0.036**	**<0.001**	0.141
no language barriers	**0.008**	**<0.001**	**0.038**	**0.002**	0.110	**<0.001**
previous expectations	**0.020**	**<0.001**	0.064	**0.005**	0.386	**0.022**
adequate information	0.414	0.227	0.338	0.255	0.098	0.468

Notes: the table refer to Rho of Spearman correlation analysis, two tails; the number refer to the *p* values, in bold are evidenced the significant *p* values

## 4. Discussion

This study is one of the first to investigate the opportunities and challenges encountered by Albanian nurses during their professional integration following their migration to Germany. The findings highlight effective workplace integration and predominantly positive experiences among the participants. Additionally, the results underscore the success of integration strategies implemented by German organizations, thus offering valuable insights and practical implications for a broader audience.

Successful integration in a new workplace, especially for migrant nurses, is a multifaceted process that requires active involvement from different stakeholders: the migrant nurses, the unit team and manager, and the organizational culture and strategy [27]. Each plays a unique role in facilitating a smooth transition and fostering a supportive environment. When these stakeholders work collaboratively, they create an environment that supports both the professional and personal adaptation of migrant nurses, thus ultimately improving patient care, workplace satisfaction, and migrant nurse retention [27].

When entering the workplace in a host country, migrant nurses actively engage in the integration process by demonstrating their adaptability, resilience, a willingness to learn, and proactive behavior [27]. Migrant nurses show a high motivation at work, which is driven by their migration aspirations [28] and the long wait process for their professional recognition [17]. Indeed, migrant nurses demonstrate a greater spirit of sacrifice, flexibility, and adaptability in the workplace as compared to their local peers, thereby often showing a willingness to work longer hours or a tendency to use less paid leave or sick days [29]. In addition, the migrant nurse's motivations are boosted from the migration drivers such as the desire for better salaries, the need to financially support their families, a better future for their children, and opportunities for professional growth [30],[31]. These factors contribute to their positive attitude toward integration and high expectations for their new professional and personal environments [27],[32].

The migrant nurses in this study likely align with findings from previous literature [27]–[29], thus suggesting that their perceptions of integration are highly positive when driven by similar motivating factors [30],[31]. This is reflected in the participants' strong agreement with statements about receiving professional respect in the workplace and having their expectations successfully met. These positive outcomes may also be supported by cultural evidence. For over three decades, Albanians have migrated to Western countries in search of better opportunities. The studies document a favorable view of Albanians' successful integration into host societies, both economically and socially [33],[34]. Albanians have demonstrated a notable capacity to integrate into new societies while preserving their cultural heritage. This resilience and adaptability likely contribute to their ability to effectively navigate and integrate into broader cultural and professional contexts [35].

Essential factors for successful integration include the openness, cultural competence, and welcoming attitudes of workplace teams and managers. Inclusivity, acceptance, and respect are fundamental to fostering a sense of belonging and supporting integration [36]. The participants in this study reported high levels of satisfaction with their autonomy, recognition of professional qualifications, and respect received from patients, families, and colleagues. Additionally, they reported a good knowledge of institutional policies and procedures, thus suggesting that the workplace environment was inclusive and supportive of migrant nurses. Indeed, mentorship programs, inclusive practices, and both formal and informal support systems can enhance collaboration, foster understanding, and create a positive work environment [27]. This, in turn, enables the full development of the migrant nurses' potential and practical abilities. While formal support mechanisms, such as training, mentoring and constructive feedback, promote competence development, informal support through friendships and peer connections positively impacts their well-being, job satisfaction, and retention [37]. Support from colleagues and managers plays a vital role in the workplace adaptation and success, thus helping migrant nurses overcome psychological challenges and enabling them to professionally excel while effectively managing the workplace demands [32]. Informal networks further boost the nurse's confidence and accelerate their professional integration [28].

Professional socialization plays a key role in helping migrant nurses learn the norms, values, and attitudes necessary to assume professional roles in host countries [29]. However, the integration process is also influenced by their prior practical experience, cultural competence, and approaches to care. Nursing practices are often shaped by societal norms; for instance, developed countries typically embrace autonomous, person-centered, or holistic nursing models, whereas many developing countries employ task-oriented, medically driven approaches [28]. These differences in professional autonomy, management styles, and expectations can present significant challenges for migrant nurses as they adapt to new care practices [28]. Additionally, discrepancies in technologies, patient demographics, and workloads between the origin and destination countries may lead to skill mismatches, thus requiring substantial time and effort for adjustment [29]. To address these challenges, the unit managers must consider tailored support programs and organizational initiatives that facilitate the adaptation of migrant nurses to the customs and expectations of the host nation and workplace [29]. Indeed, managers play a critical role in facilitating a smooth integration for migrant nurses. By setting clear expectations, offering feedback, fostering inclusivity, and addressing challenges through regular check-ins, they can enhance the nurse's confidence and job satisfaction [27]. Advocating for equal treatment and professional growth opportunities further supports integration. Managerial support, immediate supervision, and a sense of organizational value are key factors that influence retention and reduce the turnover intentions among migrant nurses [38].

Linguistic proficiency is a critical factor in the successful integration of migrant nurses. The participants in this study reported challenges in mastering the German language, particularly in understanding medical terminologies, telephone communication, interactions with physicians, patients, and caregivers, and the documentation of nursing care. While many Albanian nurses that migrate to Germany possess a basic knowledge of the language [7], they often lack proficiency in professional and medical terminologies, which emerges as a significant barrier. This limitation impedes effective workplace communication with patients, families, and colleagues, thus ultimately hindering their adaptability, career progression, and recognition of professional qualifications, respect, and autonomy. The findings of this study are consistent with the existing literature, which identifies communication challenges and cultural and social differences as significant barriers to the integration of migrant nurses into healthcare systems [39]. Similar difficulties with medical jargon and documentation have been reported among nurses who migrated to Germany [29] or international nursing students' experience in clinical settings [40]. Limited language proficiency can obstruct the nurse-patient collaboration, negatively affect the quality of care, reduce job satisfaction, and impede the integration into the healthcare system [41]. Furthermore, language barriers can prevent the formation of interpersonal relationships and social bonds in the workplace, thus hindering socialization. Studies have shown that when language difficulties exist, interactions with migrant nurses tend to be task-oriented and brief [28]. This lack of meaningful engagement can contribute to feelings of exclusion, isolation, dissatisfaction, and a reduced sense of belonging. Additionally, it may limit career advancement opportunities and create misconceptions about professional competence [28]. Therefore, addressing these linguistic and cultural challenges is essential to foster inclusivity and to ensure the successful integration of migrant nurses.

National and organizational policies play a pivotal role in determining the success of the integration process for migrant nurses. The positive integration experiences observed in this study can largely be attributed to Germany's advanced policies and strategies regarding workforce migration. The German government has established comprehensive policies that facilitate recruitment from outside the country and regulate the entry and stay of migrant workers. In terms of external recruitment, German institutions collaborate with the countries of origin to begin the initial preparation of prospective nurses [19],[21]. This preparation includes extensive support, such as intensive German language courses up to the B2 level, and clinical training to ensure that nurses acquire the theoretical and practical knowledge required to obtain a work license in Germany. Additionally, prospective nurses are provided with information about the employing institution, the country's culture, and relevant legal aspects, as well as guidance through the visa and residence permit procedures. However, the study revealed that many nurses expressed a need for additional information about the hospital structures, legal frameworks, and workplace-specific procedures to familiarize themselves with working conditions better. While this study focused on quantitative data, future research could explore the specific informational needs of migrant nurses through qualitative approaches to further enhance their decision-making processes and workplace integration.

The German government and healthcare institutions have also implemented policies to combat workplace prejudice and discrimination, promote inclusion, and ensure equity in treatments [17]. Migrant nurses receive onboarding processes similar to those of the local employees, including orientation sessions and mentorship assignments during the initial months of employment [29]. Additionally, German hospitals provide services specifically designed for migrant nurses, such as language courses, assistance with administrative tasks both inside and outside the workplace, access to housing and social networks, and support for family reunification and the employment of family members [19],[20],[29]. These measures are critical to foster successful integration and ensure the well-being of migrant nurses.

Similar to findings from other studies on migrant nurses, the participants in this research were predominantly female and married. This is in line with national statistics, as nursing in Albania, similar to other European countries, is a predominantly female profession, with women comprising approximately 80% of the workforce. Migration remains a strong aspiration for 54% of Albanians [30], and nursing is often viewed as a pathway to fulfil this aspiration rather than solely a career opportunity within the native country [42]. Traditionally, Albanian migration patterns have involved young men and women or young families with small children [7],[31], which aligns with the demographic characteristics observed in this sample. The participants in the study expressed a desire for more pre-departure information, particularly regarding family-related factors. Additionally, they highlighted the importance of the support provided by hospital structures in collaboration with German companies, which facilitated their integration by offering favorable living conditions such as housing, educational opportunities for their children, and enhanced security. These factors were reported to have positively influenced their overall integration experience.

Ultimately, nurses migrating to Germany, as in other countries, experience an integration process characterized by both opportunities and challenges across multiple dimensions. The successful integration of migrant nurses is a complex process that necessitates the coordinated efforts of national policymakers, healthcare institutions, and individual stakeholders. The migration of nurses is a significant international issue, and despite WHO recommendations for adopting a positive approach, it requires increased attention from the political structures of host countries. Stakeholders must ensure fair recruitment practices and equal treatment for migrant healthcare workers [4]. Comprehensive and inclusive strategies that address the linguistic, cultural, social, and professional needs are essential to foster the successful adaptation, well-being, and long-term retention. These efforts are critical in creating a supportive environment that allows migrant nurses to thrive and effectively contribute to the host healthcare system.

When considered in conjunction with the positive experiences reported by Albanian migrant nurses, the findings of this study suggest that other European countries may benefit from adopting policies and strategies similar to those implemented in Germany. The incorporation of effective integration measures has the potential to enhance the support for migrant nurses entering a nation's healthcare workforce, thereby promoting retention, job satisfaction, and overall workforce stability across the region.

### 4.1. Strengths and limitations

A strength of this study is the unique topic, which explores the experiences of Albanian migrant nurses in Germany. It contributes important information on the factors that facilitate successful migration and integration while highlighting the critical role of managerial and institutional support in this process. The study focuses on nurses from a Western Balkan, middle-income country—a population that has been underexplored in the existing research.

Capturing the integration experiences of this group is particularly important to compare their perspectives with those of nurses from other regions, thus offering a deeper understanding of potential cultural influences on migration and integration. Furthermore, the findings contribute to the nursing literature by addressing a gap in the study of migrant nurses from this European region. The aspects explored in this research can serve as a foundation to develop quantitative tools to evaluate the work environments and integration experiences of migrant nurses in the future.

This study has several limitations that should be acknowledged. First, concerns regarding the generalizability of the findings may arise due to the recruitment method and sample characteristics. The use of a snowball recruitment strategy, while advantageous for identifying participants in hard-to-reach populations, carries the potential for bias due to over-representation of individuals with similar characteristics and the inherent risk of disclosing personal information to others. Additionally, the cross-sectional design and the relatively small sample size limit the ability to establish causal relationships between the variables. The reliance on a convenience sample and self-reported data may introduce bias, as responses could be influenced by subjective perceptions or social desirability. Another limitation is the study's focus on quantitative data, which may not fully capture the nuances and depth of the migrant nurses' experiences. Incorporating qualitative methods in future research could provide richer insights. Furthermore, the study's findings are specific to Albanian nurses migrating to Germany, which may limit the applicability of the results to other migrant nurse populations or healthcare contexts. Despite these limitations, the study offers valuable insights into the integration experiences of a population that has been underexplored in nursing migration research.

### 4.2. Implications for practice

This study highlights Germany's effective integration strategies for migrant nurses. Essential initiatives that allow migrant nurses to flourish at the workplace encompass the following: comprehensive pre-departure training, orientation programs, mentorship initiatives, and the establishment of culturally sensitive and inclusive work environments [43]. However, for a successful migration, migrant nurses must have a clear understanding of their motivations for leaving their home country, their aspirations for mobility, and their expectations from the host country. A realistic assessment of the advantages and challenges associated with migration is crucial to ensure a positive experience. Language proficiency, particularly in medical terminology, is critical to meet the expectations and achieve successful integration. While better salaries are a key motivator for many Albanian nurses, those seeking career advancements and professional progression must prioritize linguistic skills to maximize their opportunities for professional growth. Aligning expectations with the realities of migration is essential for a successful transition and long-term integration.

High-income countries should focus on enhancing the workplace conditions to prevent the turnover rates among migrant nurses [38]. To address this issue, the host nations should implement strategies that promote integration, ensure financial equity, and provide secure long-term residency permits. These measures are essential to foster stability and retention within the migrant nursing workforce.

Meanwhile, low- and middle-income countries must prioritize retention of their nursing workforce [44]. Albanian nurse migration places a significant strain on Albania's healthcare system, which results in a shortage of skilled professionals, increased waiting times for patients, and reduced access to medical services in rural areas [45]. In addition, the migration of young and qualified professionals hinders the development of human capital, thereby slowing the growth of a nation's economy [46]. To address these challenges, the Albanian government must implement strategies to retain qualified nurses by prioritizing targeted career pathways and offering professional growth opportunities, specialization courses, skills development programs, and competitive salary improvements. Such initiatives can promote a more stable and motivated nursing workforce, thereby enhancing the overall strength of the national healthcare system [47].

## 5. Conclusions

This study explored the opportunities and challenges experienced by Albanian migrant nurses who integrated into the German healthcare system. Germany's integration framework has enabled these nurses to secure employment contracts, competitive salaries, a good quality of life, and access to continuing education, thus facilitating their adaptation to advanced procedures and modern technologies in patient care. However, this process remains complex, thereby requiring significant preparation, resilience, and responsibility to meet the professional, social, and economic standards of the host country. This research is particularly valuable for nursing professionals aspiring to migrate, thus emphasizing the importance of adequate pre-departure preparation and access to accurate information. Key areas include understanding the healthcare system, language, culture, infrastructure, and other essential aspects of life in Germany.

Given the critical role of nurses in healthcare systems, where the quality of care reflects national standards, further research is recommended to examine the integration processes of Albanian nurses across other European countries and how these processes are implemented. Additionally, future studies need to continue monitoring the nurse migration flux in Albania and examine the effectiveness of governmental and institutional actions to retain nurses in the country and enhance patient care. Future studies should focus on identifying and addressing potential barriers to integration while fostering opportunities for collaboration between the origin and destination countries. Such research can contribute to academic, political, and social frameworks that enhance the migration experience and ensure a smoother, more effective integration process for migrant nurses.

## Use of AI tools declaration

The authors declare they have used Artificial Intelligence (AI) tools, specifically ChatGPT and Grammarly to correct language errors.
